# Supplementary material for: Modeling glioblastoma heterogeneity as a dynamic network of cell states
Source: Mol Syst Biol. 2021 Sep 16;17(9):e10105. doi: 10.15252/msb.202010105 (PMC8444284; doi:10.15252/msb.202010105)
Supplement: Supplementary file 6 — Source Data for Figure 5 [file MSB-17-e10105-s004.zip › Figure5A_sourcedata/GSEA_3071/nosigdb_stateVI.GseaPreranked.1622795169056/LARSSON_STATE_4.html]

Details for gene set LARSSON\_STATE\_4[GSEA]

|  || Dataset | state63071 |
| Phenotype | NoPhenotypeAvailable |
| Upregulated in class | na\_neg |
| GeneSet | LARSSON\_STATE\_4 |
| Enrichment Score (ES) | -0.5847843 |
| Normalized Enrichment Score (NES) | -2.720042 |
| Nominal p-value | 0.0 |
| FDR q-value | 0.0 |
| FWER p-Value | 0.0 |
Table: GSEA Results Summary

  

Fig 1: Enrichment plot: LARSSON\_STATE\_4      
 Profile of the Running ES Score & Positions of GeneSet Members on the Rank Ordered List

  

| PROBE | GENE SYMBOL | GENE\_TITLE | RANK IN GENE LIST | RANK METRIC SCORE | RUNNING ES | CORE ENRICHMENT || 1 | CCNB1 |  |  | 181 | 0.620 | -0.0359 | No |
| 2 | PTTG1 |  |  | 344 | 0.514 | -0.0691 | No |
| 3 | RAN |  |  | 712 | 0.394 | -0.1624 | No |
| 4 | UBE2S |  |  | 974 | 0.349 | -0.2272 | No |
| 5 | BIRC5 |  |  | 1667 | 0.263 | -0.4146 | No |
| 6 | MKI67 |  |  | 1825 | -0.253 | -0.4525 | No |
| 7 | SGO2 |  |  | 1842 | -0.255 | -0.4510 | No |
| 8 | HMGN2 |  |  | 1857 | -0.258 | -0.4487 | No |
| 9 | MND1 |  |  | 1943 | -0.270 | -0.4661 | No |
| 10 | RFC2 |  |  | 2044 | -0.284 | -0.4874 | No |
| 11 | GTSE1 |  |  | 2073 | -0.291 | -0.4883 | No |
| 12 | SGO1 |  |  | 2249 | -0.323 | -0.5296 | No |
| 13 | CENPM |  |  | 2287 | -0.330 | -0.5321 | No |
| 14 | ORC6 |  |  | 2343 | -0.342 | -0.5394 | No |
| 15 | FANCI |  |  | 2411 | -0.356 | -0.5497 | No |
| 16 | PBK |  |  | 2412 | -0.356 | -0.5412 | No |
| 17 | MCM5 |  |  | 2424 | -0.359 | -0.5358 | No |
| 18 | UHRF1 |  |  | 2549 | -0.388 | -0.5613 | No |
| 19 | SMC4 |  |  | 2634 | -0.408 | -0.5751 | Yes |
| 20 | ZWINT |  |  | 2641 | -0.410 | -0.5670 | Yes |
| 21 | STMN1 |  |  | 2646 | -0.411 | -0.5584 | Yes |
| 22 | ASF1B |  |  | 2704 | -0.427 | -0.5642 | Yes |
| 23 | CDC45 |  |  | 2776 | -0.448 | -0.5734 | Yes |
| 24 | MCM7 |  |  | 2808 | -0.460 | -0.5712 | Yes |
| 25 | CENPF |  |  | 2842 | -0.472 | -0.5692 | Yes |
| 26 | KIF15 |  |  | 2855 | -0.478 | -0.5612 | Yes |
| 27 | SMC2 |  |  | 2864 | -0.483 | -0.5520 | Yes |
| 28 | UBE2T |  |  | 2868 | -0.484 | -0.5414 | Yes |
| 29 | NCAPG |  |  | 2951 | -0.514 | -0.5521 | Yes |
| 30 | TMPO |  |  | 3022 | -0.552 | -0.5586 | Yes |
| 31 | CHAF1A |  |  | 3023 | -0.552 | -0.5455 | Yes |
| 32 | H2AFX |  |  | 3024 | -0.553 | -0.5323 | Yes |
| 33 | GMNN |  |  | 3046 | -0.561 | -0.5249 | Yes |
| 34 | MCM3 |  |  | 3064 | -0.570 | -0.5161 | Yes |
| 35 | RRM1 |  |  | 3070 | -0.574 | -0.5039 | Yes |
| 36 | CENPU |  |  | 3084 | -0.578 | -0.4938 | Yes |
| 37 | DEK |  |  | 3098 | -0.583 | -0.4836 | Yes |
| 38 | GINS2 |  |  | 3119 | -0.598 | -0.4750 | Yes |
| 39 | CENPK |  |  | 3134 | -0.610 | -0.4644 | Yes |
| 40 | E2F1 |  |  | 3140 | -0.613 | -0.4512 | Yes |
| 41 | MYBL2 |  |  | 3196 | -0.660 | -0.4510 | Yes |
| 42 | NUSAP1 |  |  | 3199 | -0.663 | -0.4358 | Yes |
| 43 | LMNB1 |  |  | 3246 | -0.711 | -0.4318 | Yes |
| 44 | MCM4 |  |  | 3254 | -0.715 | -0.4168 | Yes |
| 45 | EZH2 |  |  | 3282 | -0.741 | -0.4067 | Yes |
| 46 | DHFR |  |  | 3303 | -0.765 | -0.3941 | Yes |
| 47 | DUT |  |  | 3307 | -0.769 | -0.3767 | Yes |
| 48 | RRM2 |  |  | 3317 | -0.783 | -0.3606 | Yes |
| 49 | TYMS |  |  | 3333 | -0.798 | -0.3459 | Yes |
| 50 | BRCA1 |  |  | 3335 | -0.802 | -0.3271 | Yes |
| 51 | TMSB15A |  |  | 3358 | -0.824 | -0.3137 | Yes |
| 52 | MCM10 |  |  | 3360 | -0.825 | -0.2944 | Yes |
| 53 | RAD51AP1 |  |  | 3375 | -0.848 | -0.2781 | Yes |
| 54 | PCLAF |  |  | 3387 | -0.871 | -0.2605 | Yes |
| 55 | CDK1 |  |  | 3396 | -0.883 | -0.2418 | Yes |
| 56 | FBXO5 |  |  | 3413 | -0.919 | -0.2244 | Yes |
| 57 | HMGB2 |  |  | 3414 | -0.922 | -0.2026 | Yes |
| 58 | PCNA |  |  | 3415 | -0.922 | -0.1807 | Yes |
| 59 | TOP2A |  |  | 3457 | -1.020 | -0.1679 | Yes |
| 60 | ESCO2 |  |  | 3479 | -1.062 | -0.1485 | Yes |
| 61 | CLSPN |  |  | 3499 | -1.115 | -0.1274 | Yes |
| 62 | HIST1H1E |  |  | 3504 | -1.125 | -0.1018 | Yes |
| 63 | HIST1H1C |  |  | 3509 | -1.145 | -0.0757 | Yes |
| 64 | ATAD2 |  |  | 3534 | -1.234 | -0.0531 | Yes |
| 65 | HIST1H1B |  |  | 3592 | -1.679 | -0.0291 | Yes |
| 66 | HIST1H1D |  |  | 3596 | -1.757 | 0.0118 | Yes |
Table: GSEA details [plain text format]

  

Fig 2: LARSSON\_STATE\_4: Random ES distribution      
 Gene set null distribution of ES for **LARSSON\_STATE\_4**
